# Sarcopenia and Nutrition on YouTube: A Content Quality and Reliability Assessment

**DOI:** 10.3390/nu18040619

**Published:** 2026-02-13

**Authors:** Carmen Trost, Richard Crevenna, Jacob Heisinger, Domenik Popp, Annemarie Perl, Eva-Maria Marchard, Stephan Heisinger

**Affiliations:** 1 Department Teaching Center, Medical University of Vienna, 1090 Vienna, Austria; carmen.trost@meduniwien.ac.at; 2 Department of Physical Medicine, Rehabilitation and Occupational Medicine, Medical University of Vienna, 1090 Vienna, Austria; richard.crevenna@meduniwien.ac.at; 3 Department of Pediatrics, Krankenhaus der Barmherzigen Brüder Eisenstadt, 7000 Eisenstadt, Austria; jacob.heisinger@gmx.at; 4 Department of Orthopedics and Trauma-Surgery, University Hospital Neunkirchen, 2620 Neunkirchen, Austria; domenik.popp@meduniwien.ac.at; 5 Omanda AG, 3072 Ostermundigen, Switzerland; annemarie.perl@omanda.ch; 6 Department Internal Medicine, Zentrum für Experimentelle und Ultraschallgestützte Studien, Federal Clinic Baden-Mödling, 2340 Baden-Modling, Austria; eva-maria.marchard@gmx.at; 7 Department of Orthopedics and Trauma Surgery, Medical University of Vienna, 1090 Vienna, Austria

**Keywords:** sarcopenia, nutrition, digital health, YouTube, health literacy, older adults

## Abstract

Background/Objectives: Sarcopenia is a prevalent age-related condition strongly influenced by protein intake. This study assessed the quality, evidence base, and practical utility of YouTube videos on nutrition and sarcopenia. Methods: A structured YouTube search (We searched YouTube in April 2024 using the term ‘sarcopenia diet) identified 41 eligible videos. Three trained raters independently assessed each video using the Global Quality Scale (GQS), DISCERN, JAMA benchmarks, subjective impression ratings, technical quality indicators, and additional binary variables. Interrater reliability was examined using intraclass correlation coefficients (ICCs) and Fleiss’ Kappa. Reviewer comments were analyzed qualitatively using Mayring’s structured content analysis. Results: Overall video quality varied substantially. ICCs indicated moderate to high agreement for DISCERN (0.698), JAMA (0.702), and subjective impression ratings (0.779), but minimal agreement for sound and video quality. Fleiss’ Kappa showed moderate agreement for scientific soundness (κ = 0.522) and advertisement content (κ = 0.385), while agreement was low for health-related risks and dietary recommendations. Qualitative analysis identified frequent concerns regarding insufficient scientific evidence, vague or impractical protein guidance, limited relevance for older adults, and personal bias; positive features were less common. Conclusions: YouTube nutrition content on sarcopenia shows substantial variability and frequent deficits in evidence-based quality and practical relevance. While some videos provide useful introductory information, many are of limited value for lay audiences. Strengthening digital health literacy and expanding expert-driven, evidence-based online resources are essential to support informed decision-making and preventive strategies.

## 1. Introduction

Sarcopenia is a progressive skeletal muscle disorder characterized by age-related loss of muscle mass and function and is associated with falls, disability, loss of independence, reduced quality of life, and increased mortality [1,2,3]. As pharmacological treatment options remain limited, preventive strategies, particularly adequate nutrition and regular physical activity, are central to mitigating age-related muscle loss [2,4,5]. High-quality protein intake and nutrients such as vitamin D and leucine support muscle protein synthesis and are key components of nutritional management in older adults [3,4,5,6,7,8,9,10,11,12,13].

Parallel to growing scientific interest in nutritional strategies for sarcopenia, online platforms have become widely used sources of health information. YouTube (Google LLC, Mountain View, CA, USA) in particular is frequently accessed by patients and caregivers seeking dietary guidance due to its accessibility, visual format, and wide reach. However, the accuracy and evidence base of user-generated content remain highly variable. Prior research has shown that the informational quality of health-related YouTube videos is inconsistent and that popularity metrics do not reliably reflect scientific accuracy [1]. Older adults, who are disproportionately affected by sarcopenia, increasingly rely on online material for health-related decision-making, despite variations in digital health literacy.

Despite substantial scientific literature addressing nutrition and sarcopenia, illustrated by more than 1300 related publications indexed on PubMed, no studies to date have assessed the quality of YouTube videos providing nutritional guidance on sarcopenia, despite the platform’s widespread use. This gap is striking given the platform’s growing role in public health communication and the increasing tendency of patients to consult online videos for disease management and dietary decision-making.

This study aimed to evaluate the quality, evidence base, and practical relevance of YouTube videos providing nutritional information on sarcopenia, using validated assessment tools and interrater reliability metrics.

## 2. Materials and Methods

A user-based YouTube search was conducted, intended to reflect how a lay person might search for nutritional information related to sarcopenia. The term “sarcopenia Diet” was selected because it represents a straightforward phrase likely to be used by patients or caregivers seeking dietary guidance. The search was conducted on a single day (8 April) using an incognito browser window while logged out of any Google account to minimize personalization, with language and location set to English and results displayed according to YouTube’s default relevance-based ranking. A total of *n* = 60 videos were initially screened, of which *n* = 41 met the inclusion criteria. Videos were excluded if they were not in English, did not primarily address dietary or nutritional aspects of sarcopenia, or if they were excessively long (>30 min) or fragmented in a way that prevented consistent evaluation. Additional exclusions included overly scientific content, such as academic lectures or experimental animal studies, and videos that focused exclusively on general muscle building, celebrity opinions (e.g., Joe Rogan), or exercise-only without a nutritional context.

Each included video was assessed independently by three raters: two raters were licensed nutrition professionals (AP, EMM), and one was a pediatrician clinician with postgraduate training in clinical nutrition (JH). All raters received standardized instructions and completed calibration exercises before data extraction.

This study analyzed publicly available YouTube material and involved no human participants; therefore, ethics approval was not required in accordance with local regulations.

To evaluate the quality and informational value of the videos, several validated tools and criteria were applied. The Global Quality Scale (GQS) was used to provide an overall rating of video quality, ranging from 1 (poor) to 5 (excellent), with higher scores indicating higher overall quality. Reliability was evaluated using the validated 5-item DISCERN short form (higher scores indicating higher reliability). The Journal of the American Medical Association (JAMA) benchmark criteria (0–4, higher indicating greater transparency and accuracy) were applied [14,15]. A subjective overall impression of each video was captured on a scale from 1 (excellent) to 5 (very poor). Technical quality regarding audio and visual clarity was likewise rated from 1 (excellent) to 5 (insufficient). Moreover, several binary indicators were recorded, including the presence of advertisements, background information, potential health hazards, explicit mentions of such hazards, the scientific basis of the content, and the appropriateness of the recommendations provided. Videos were classified as scientifically sound if their statements aligned with evidence-based nutritional recommendations for sarcopenia, avoided unsupported claims, and showed consistency with recognized clinical guidelines.”

Quantitative data were analyzed using SPSS version 29.0.0. (IBM Corp., Armonk, NY, USA) and R version 4.3.2. (R Foundation for Statistical Computing, Vienna, Austria) Descriptive statistics (mean, median, standard deviation, range) were calculated for each rating scale across raters. Interrater agreement was assessed using the Intraclass Correlation Coefficient (ICC, two-way random model, absolute agreement) which was interpreted using the Koo criteria: above 0.90 as excellent, 0.75–0.90 as good, 0.50–0.75 as moderate, and 0.50 as poor [16]. For Fleiss’ Kappa, binary rating significance was set at ρ-value < 0.05. The Fleiss’ Kappa was interpreted according to Landis and Koch: 0.00–0.20 low agreement, 0.21–0.40 moderate agreement, 0.41–0.60 average agreement, 0.61–0.80 substantial agreement, and 0.81–1.00 nearly perfect agreement [17].

In addition, a qualitative content analysis of free-text comments from raters was conducted qualitatively using Mayring’s structured summarizing approach [18]. Two researchers independently coded the material using an inductive, text-driven approach, deriving all categories directly from the content of the raters’ comments and resolving discrepancies through discussion.

## 3. Results

Mean scores for the DISCERN scale ranged from 1.54 to 2.66, with all raters showing comparable variability (SD 0.99–1.20) and median values between 1 and 2. The observed ranges extended from 0 to 4 for one rater, while two others reached 0–5. On the JAMA scale, mean scores varied between 1.83 and 2.27, with consistently moderate variability (SD 1.03–1.28) and a median of 2 for all raters. The score range was from 1 to 4 or 0 to 4 depending on the rater. On the Opinion scale, the mean scores were markedly higher, spanning 2.88–3.34. The SDs were slightly lower (0.87–1.09), with medians of 3 or 4 and observed score ranges of 1–5 or 1–4. For the Global Quality Scale (GQS), mean scores ranged from 2.90 to 3.66, with medians of 3–4 and standard deviations around 1, indicating variability in perceived overall video quality across raters.

These results indicate that raters tended to assign higher ratings on the Opinion scale than on DISCERN or JAMA. All scales demonstrated moderate consistency across raters, as evidenced by similar SDs. The GQS showed a comparable pattern of dispersion, suggesting that global assessments of video quality also varied notably among reviewers. 

### 3.1. Descriptive Statistics

However, some inter-rater variation in central tendency and range was observed, particularly for the DISCERN scale (Table 1).

### 3.2. Interrater Reliability

To assess the consistency of the three independent reviewers, ICC for ordinal-scale items and Fleiss’ Kappa for binary items, based on ratings from 41 videos.

### 3.3. ICC Results for Ordinal Scales

As shown in Table 2, the ICC values for the main ordinal rating scales: GQS total, DISCERN total, JAMA total, Subjective Opinion, and the technical evaluations of sound and video quality. The results indicate moderate interrater agreement for GQS (ICC = 0.723, 95% CI [0.505–0.848]), DISCERN (ICC = 0.698; CI [0.393–0.845]), JAMA (ICC = 0.702; CI [0.504–0.830]), and especially for Subjective Opinion (ICC = 0.779; CI [0.627–0.875]), all of which were statistically significant. In contrast, agreement for sound quality (ICC = 0.083; CI [−0.078–0.283]) and video quality (ICC = −0.16; CI [−0.116–0.129]) was non-significant, suggesting considerable variability between raters for these more subjective technical aspects.

### 3.4. Fleiss’ Kappa

In Table 3, the Fleiss’ Kappa values show statistics for dichotomous variables. For Fleiss’ Kappa, *p*-values were obtained using the standard asymptomatic test implemented in SPSS. Average agreement was observed for the item Scientifically sound (κ = 0.522, *p* < 0.001); while moderate agreement was found for Advertisement (κ = 0.385, *p* < 0.001) and Background information (κ = 0.312, *p* < 0.001), Agreement was weaker for Potential health hazards (κ = 0.261, *p* = 0.004) and Recommendations (κ = 0.194, *p* = 0.027) although both reached statistical significance. No significant agreement was observed for the item Potential health hazards mentioned (κ = 0.114, *p* = 0.205), indicating notable discrepancies between raters in identifying whether these risks were explicitly stated. In addition to agreement statistics, the prevalence of each binary indicator was calculated. Scientifically sound content was judged present in 38 videos (92.7%), advertisement took place in 22 videos (53.7%). Background information was present in most videos (40/41; 97.6%). Potential health hazards were identified in 9 videos (22.0%). Recommendations were included in 36 videos (87.8%), and potential health hazards were explicitly mentioned in 16 videos (39.0%).

These findings highlight that while content-related assessments (e.g., evidence base, recommendations) showed moderate to good agreement, technical or nuanced binary classifications were more prone to variability among raters.

### 3.5. Exploratory Associations with Video Popularity

To explore whether video characteristics influenced quality ratings, two additional analyses were performed. First, Spearman’s rank correlations between view counts and quality indicators (GQS, DISCERN, JAMA, Opinion) showed no meaningful associations (ρ = –0.076 to 0.253), indicating that more frequently viewed videos were not of higher scientific quality (Supplementary S3). Second, a Mann–Whitney U test compared quality scores between videos uploaded before versus after 2020. No significant differences were found for any scale (GQS: U = 191.5, *p* = 0.946; DISCERN: U = 181, *p* = 0.839; JAMA: U = 246.5, *p* = 0.115; Opinion: U = 214, *p* = 0.505), suggesting that content quality did not differ by upload period (Supplementary S3). Together, these exploratory analyses indicate that neither video popularity nor publication period was associated with higher reliability or evidence-based nutritional content.

### 3.6. Qualitative Analysis of Rater Comments

**Figure 1:**
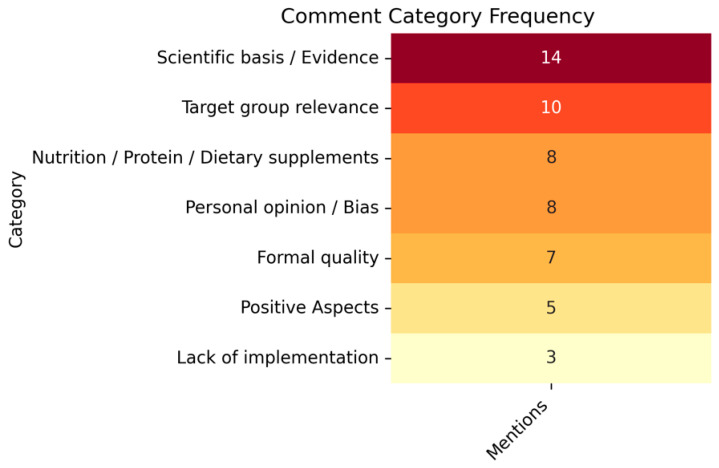
Heatmap illustrating the frequency of seven comment categories derived from qualitative analysis. Darker shades indicate higher mention counts, with scientific evidence and target group relevance most frequently cited.

In addition to the quantitative evaluation, an inductive qualitative content analysis of the rater comments was conducted following Mayring’s summarizing content analysis approach (16). Emerging themes were categorized and grouped based on their frequency of occurrence (Figure 1). Color intensity reflects the number of mentions, with darker shades indicating higher frequency (range 3–14). The most frequently mentioned categories concerned Scientific bias/Evidence (*n* = 14), Target group relevance (*n* = 10), and Nutrition/Protein/Dietary supplements (*n* = 8), suggesting that raters prioritized content accuracy and relevance for the intended audience. Formal shortcomings were also noted (Formal quality, *n* = 7) but appeared to little to influence on the substantive content. Less frequently mentioned themes included Lack of implementation (*n* = 3) and Positive aspects (*n* = 5), indicating a general critical tone in the comment corpus.

The qualitative analysis of the comments revealed several recurring themes. Many videos were criticized for lacking an adequate base, either due to missing source citations or because claims were primarily grounded in personal opinion rather than scientific literature. Target group specificity emerged as a central issue, with content frequently perceived as either too complex for lay audiences or too superficial for healthcare professionals. Nutritional aspects, particularly protein intake, were commonly addressed; however, recommendations were often vague or lacked practical guidance. Formal shortcomings such as video quality, length, and structural coherence were also noted, although these aspects appeared to be weighted less heavily than content-related deficiencies. Conversely, some videos received positive feedback, particularly for the use of visual aids, clear messaging, and patient-centered language.

## 4. Discussion

Social media has become an indispensable source of information, a trend repeatedly demonstrated across different populations. The phenomenon of online health information seeking has been well documented in the literature. A systematic review by Jia et al. reported that individuals increasingly use the internet to seek health information, with studies identifying common behavioral patterns and a range of influencing factors such as literacy levels, demographic characteristics, and accessibility barriers and facilitators (e.g., online communities, privacy features, and real-time interaction) [19]. These findings underscore that seeking health information online has become a significant component of consumer health behavior across populations. Similarly, Zhao and Zhang depicted research on how consumers utilize social media for both health information and emphasized both the potential benefits, such as peer support and easier access to content, and concerns about information quality and authoritative credibility [20]. More recent scoping reviews further highlight that social media platforms and general internet sources are common channels for health information, but also that specific platform characteristics influence engagement, perceived credibility, and trust [21].

While these studies provide useful context, they also highlight a consistent problem: online health information is highly variable, and users may struggle to differentiate evidence-based material from misleading or anecdotal content. In this light, our findings offer an applied example of how these broader concerns manifest within nutrition-related sarcopenia content on YouTube. Similarly, de Stefano et al. (2026) note that complex nutritional issues, such as protein quality and micronutrient adequacy in vegetarian diets, are often difficult to communicate to non-expert audiences, which can lead to oversimplification in popular media [22].

Across the reviewed literature, digital platforms are portrayed as a double-edged tool within health communication. On the one hand, the internet is described as an optimal medium for disseminating health information due to its low cost, broad accessibility, and ability to reach large audiences without geographic limitations. YouTube in particular offers considerable potential for delivering content in scale. Our findings align with those of Akyol and Karahan (2020) ([1]), who similarly reported moderate overall informational quality and substantial variability across YouTube videos on sarcopenia. While their assessment focused primarily on general disease information, our study extends these insights by specifically evaluating nutrition-related content, revealing comparable inconsistencies in evidence-based guidance and practical applicability [1]. However, these theoretical advantages do not necessarily translate into high-quality information, as concerns about the scientific accuracy and reliability of user-generated content are repeatedly emphasized. In the absence of peer-review mechanisms, YouTube videos often exhibit only moderate or poor informational accuracy, and popularity indicators such as views or likes are not reliable markers of evidence quality [1,23]. The literature further warns that misleading or inappropriate advice may harm users if exercises or recommendations do not align with specific clinical conditions. Given that lay audiences frequently struggle to evaluate the credibility of online material, several authors call for stronger professional presence in digital spaces to promote evidence-based guidance and support informed decision-making [12].

Falls, frailty, and healthcare costs form a self-reinforcing cycle in older adults: sarcopenia and frailty increase fall risk, falls frequently result in costly hospitalizations and surgical interventions, and subsequent immobility further accelerates muscle loss and functional decline, culminating in a “catabolic crisis” [3,7]. This cycle is particularly problematic in aging societies, where the number of affected individuals is rapidly increasing and loss of independence places growing demands on health and social care systems [9,10]. Although preventive strategies such as targeted nutrition and physical activity interventions are cost-effective, they are often implemented too late or insufficiently [3,10]. In this context, digital platforms such as YouTube may substantially influence health behaviors and decision-making related to sarcopenia prevention. However, metrics such as upload date, popularity indicators, or correlations between rating dimensions were not examined because the study prioritized content quality over platform-driven engagement characteristics [12,24,25].

In view of our results on the variable quality of sarcopenia-related YouTube content, the implications for preventive care and public health are substantial. Nutrition and exercise interventions have demonstrated measurable improvements in protein intake, physical performance, and muscle mass in older adults, underscoring the potential of online platforms for preventive strategies [12]. Individuals frequently struggle to assess the credibility of digital health information, making them vulnerable to misleading content; professionally produced materials are consistently more accurate than user-generated videos [1]. Across studies, limited digital health literacy is identified as a key risk factor for misinformation, underscoring the need for accessible, evidence-based online resources [1]. These insights align with well-established evidence that adequate protein intake and targeted nutrition are central modifiable factors for mitigating age-related declines in muscle mass and functional capacity [3]. Our findings further indicate that accessibility alone does not guarantee informational quality: the absence of peer review on platforms such as YouTube allows inaccurate, overly simplified, or anecdotal content to circulate widely. The moderate interrater agreement observed for the Global Quality Scale (GQS) in our study reflects this heterogeneity, suggesting that even global impressions of video quality vary substantially between raters. Consistent with previous evaluations of online health information quality, the present study highlights the need for expert-curated, evidence-based digital resources to support public health efforts in sarcopenia prevention and strengthen the reliability of nutrition-related online communication.

Several limitations of this study should be acknowledged. First, the analysis reflects a single time point and therefore captures only a momentary selection of YouTube content, even though the platform is highly dynamic and continuously changing in terms of available videos, ranking algorithms and user interactions. This restricts the reproducibility and long-term generalizability of the findings. Second, despite the use of structured search terms and predefined inclusion criteria, the selection process was influenced by platform-specific factors outside the researchers’ control. A notable number of videos had to be excluded because they were excessively long or addressed relevant nutritional aspects only towards the end, making a consistent and comparable evaluation impossible. Third, although validated tools such as GQS, DISCERN, JAMA, and subjective rating scales were applied, part of the assessment inevitably relied on subjective judgment, as reflected in the variability of interrater agreement across scales. Fourth, variables such as upload date, popularity metrics, or relationships among rating indicators were not analyzed, as the study design focused on content quality rather than engagement patterns or temporal trends. Finally, the evaluation focused exclusively on English-language videos and on content related to nutrition and sarcopenia, which limits the extent to which the results can be transferred to other languages, cultural contexts or broader health topics. Taken together, these limitations should be considered when interpreting the findings, although they also reflect the inherent methodological challenges of analyzing health information on open, unregulated video platforms.

## 5. Conclusions

This study reveals substantial variability and frequent shortcomings in the evidence-based quality of YouTube videos including in our analysis. While some videos provided clear and relevant information, many lacked scientific accuracy, consistent referencing, or practical applicability. Interrater agreement was moderate for validated quality indicators but low for technical aspects, and qualitative findings highlighted recurring concerns regarding scientific rigor, practical guidance, and personal bias. Given the growing role of online platforms in health information seeking, particularly among older adults, our findings underscore the need for further efforts to enhance the quality and clarity of publicly available digital health information related to sarcopenia.

## Figures and Tables

**Table 1:** The table presents descriptive statistics for assessments using four scales (GQS, DISCERN, JAMA, Opinion) conducted by three independent raters. For each combination of scale and rater, the mean, median, standard deviation (SD), and observed range are reported.

Scale	Rater	Mean	Median	SD	Range
GQS	R1	3.66	4	0.965	1–5
GQS	R2	2.90	3	0.970	1–4
GQS	R3	3.39	4	1.07	1–5
DISCERN	R1	1.54	1	1.16	0–4
DISCERN	R2	2.37	2	1.20	0–5
DISCERN	R3	2.66	2	0.99	0–5
JAMA	R1	1.93	2	1.03	0–4
JAMA	R2	1.83	2	1.28	0–4
JAMA	R3	2.27	2	1.03	1–4
Opinion	R1	3.34	4	0.91	1–5
Opinion	R2	2.88	3	0.87	1–4
Opinion	R3	3.17	3	1.09	1–5

**Table 2:** This table shows that the interrater reliability was moderate to high for GQS, DISCERN, JAMA, and Opinion ratings, but substantially lower and non-significant for the technical evaluations of sound and video quality.

Scale	ICC Average	95% CI	*n*	Significance
GQS	0.723	[0.505–0.848]		sig.
DISCERN	0.698	[0.393–0.845]	41	sig
JAMA	0.702	[0.504–0.830]	41	sig
Opinion	0.779	[0.627–0.875]	41	sig
Sound	0.083	[−0.078–0.283]	41	ns
Video	−0.16	[−0.116–0.129]	41	ns

**Table 3:** This table shows that Fleiss’ Kappa indicated moderate agreement for scientific accuracy, advertisement, and background information, but only low or non-significant agreement for health hazard-related items and recommendations. The value *n* refers to the number of articles in which the category was identified.

Scale	Kappa	*p*-Value	*n* (%)
Scientifically sound	0.522	*p* < 0.001	38 (92.7%)
Advertisement	0.385	*p* < 0.001	22 (53.7%)
Background info	0.312	*p* < 0.001	40 (97.6%)
Potential health hazards	0.261	*p* = 0.004	9 (22.0%)
Recommendations	0.194	*p* = 0.027	36 (87.8%)
Potential health hazards mentioned	0.114	*p* = 0.205 (n.s.)	16 (39.0%)

## Data Availability

The raw data supporting the findings of this study are available in the Supplementary Materials (Supplementary S1). Qualitative rater comments were not shared because they may contain identifiable phrasing and cannot be anonymized without altering their content; therefore, they are excluded from the public dataset.

## Supplementary Materials

The following supporting information can be downloaded at: https://www.mdpi.com/article/10.3390/nu18040619/s1, Supplementary S1: Raw Data; Supplementary S2: Videos; Supplementary S3: Exploratory Associations.

## Abbreviations

The following abbreviations are used in this manuscript:

DISCERN	standardized quality instrument for health information
GQS	Global Quality Scale
ICC	Intraclass Correlation Coefficient
JAMA	Journal of the American Medical Association Benchmark Criteria
